# Commercial Opportunity or Addressing Unmet Needs—Loop-Mediated Isothermal Amplification (LAMP) as the Future of Rapid Diagnostic Testing?

**DOI:** 10.3390/diagnostics14171845

**Published:** 2024-08-24

**Authors:** Jelle J. Feddema, Kenneth D. S. Fernald, Bart J. F. Keijser, Jasper Kieboom, Linda H. M. van de Burgwal

**Affiliations:** 1 Athena Institute, Vrije Universiteit Amsterdam, De Boelelaan 1085, 1081 HV Amsterdam, The Netherlands; k.d.s.fernald@vu.nl (K.D.S.F.); l.h.m.vande.burgwal@vu.nl (L.H.M.v.d.B.); 2 TNO Healthy Living and Work, Microbiology and Systems Biology, Sylviusweg 71, 2333 BE Leiden, The Netherlands; bart.keijser@tno.nl (B.J.F.K.); jasper.kieboom@tno.nl (J.K.)

**Keywords:** molecular diagnostics, research, innovation, technology forecasting, societal impact

## Abstract

Loop-Mediated Isothermal Amplification (LAMP) technology is emerging as a rapid pathogen testing method, potentially challenging the RT-PCR “gold standard”. Despite recent advancements, LAMP’s widespread adoption remains limited. This study provides a comprehensive market overview and assesses future growth prospects to aid stakeholders in strategic decision-making and policy formulation. Using a dataset of 1134 LAMP patent documents, we analyzed lifecycle and geographic distribution, applicant profiles, CPC code classifications, and patent claims. Additionally, we examined clinical developments from 21 curated clinical trials, focusing on trends, geographic engagement, sponsor types, and the conditions and pathogens investigated. Our analysis highlights LAMP’s potential as a promising rapid pathogen testing alternative, especially in resource-limited areas. It also reveals a gap between clinical research, which targets bacterial and parasitic diseases like malaria, leishmaniasis, and tuberculosis, and basic research and commercial efforts that prioritize viral diseases such as SARS-CoV-2 and influenza. European stakeholders emphasize the societal impact of addressing unmet needs in resource-limited areas, while American and Asian organizations focus more on research, innovation, and commercialization.

## 1. Introduction

Recent outbreaks of infectious diseases have stimulated renewed attention from governments and industries towards the field of epidemiological surveillance and outbreak management. The successful implementation of surveillance and outbreak management strategies heavily relies on the early identification of (novel) pathogens [1]. Consequently, when confronted with an infectious disease outbreak, an urgent demand arises for diagnostic tests that are both cost-effective and highly efficient, capable of being rapidly scaled up to cover large populations.

Molecular diagnostics based on Nucleic Acid Amplification Technologies (NAATs) have gained increasing importance in pathogen detection. Polymerase chain reaction (PCR) has emerged as the gold standard, also during the COVID-19 pandemic [2]. PCR is preferred over other diagnostic techniques due to its exceptional specificity and sensitivity, enabling the detection of even minute amounts of pathogenic genetic material [3].

Despite the advantages offered by PCR, several drawbacks are associated with its use. A primary limitation is the requirement for specialized laboratory equipment that cannot be easily exchanged between systems (e.g., reagents and plates) and a controlled laboratory environment with trained personnel. This renders PCR relatively costly and less accessible in resource-limited settings. Moreover, during the recent COVID-19 pandemic, this also led to scarcity of equipment and bottlenecks in testing capacity. Additionally, although PCR offers faster results than traditional culture-based methods, it still takes several hours to generate results, posing a significant disadvantage when prompt decisions are needed during outbreak responses.

Limitations associated with PCR have stimulated the advancement of non-PCR-based NAATS, amongst which loop-mediated isothermal amplification (LAMP) has emerged as the leading, most effective, rapid, and commercially viable alternative [3,4]. This technique, based on strand displacement, was discovered, and published by Notomi et al. in 2000 [5] and has witnessed a significant surge in popularity over the years, as evidenced by the exponential growth in LAMP-related publications and citations. In comparison to PCR, LAMP exhibits slightly lower sensitivity and diagnostic accuracy [4,6]. However, LAMP techniques require less elaborate sample preparation, eliminate the need for thermal cycling, and offer shorter turnaround times [4,7]. Current state-of-the-art LAMP solutions entail Point-of-Care (PoC) diagnostics that can render results in as quick as 12 min [8].

The characteristics of LAMP techniques make them particularly advantageous for settings with limited resources. The affordability and simplicity of LAMP techniques enable screening to be conducted in regions lacking laboratories, trained personnel, or even electricity or instruments [9,10,11]. Such circumstances typically present themselves in regions that bear a high burden of neglected tropical diseases (NTDs), and for which inadequate diagnostics have been identified as a critical weakness, as noted by Bharadwaj et al. (2021) [12] and Feddema et al. (2018) [13]. This is precisely where LAMP techniques are believed to demonstrate their greatest value.

Moreover, the extensive scale of the COVID-19 pandemic has revealed that diagnostic bottlenecks are not merely limited to the developing world [14]. Therefore, while LAMP techniques are frequently positioned as diagnostic tools for resource-limited settings, their widespread deployment during the COVID-19 pandemic suggests their added value in other parts of the world as well, in the form of PoC testing during sudden outbreaks.

Technological development in LAMP diagnostics has rapidly grown over the years, yet widespread adoption of its applications is still limited. Moreover, a comprehensive understanding of the LAMP diagnostic market has yet to be established. Existing studies of the field primarily focus on comparison of technologies and its applications [3,4,15]. Previous studies have failed to provide insights into the current market landscape of innovation, global research efforts, and development stages. Given the evolving landscape, developments, and concerns within the field of LAMP diagnostics, a comprehensive overview of the market and an assessment of its future growth prospects are necessary to assist stakeholders such as researchers, industry professionals, and policy makers in strategic decision-making and policy development. Hence, the purpose of this study is to offer a detailed description of the present state of the LAMP diagnostics innovation pipeline. To accomplish this, we have constructed a novel and distinctive dataset based on patent documents and clinical trials.

## 2. Materials and Methods

The objective of this study is to assess and map the technological developments and innovation landscaping regarding rapid detection of pathogens based on Loop-Mediated Isothermal Amplification (LAMP). Relevant datasets derived from both patent and clinical trial databases were studied to gain insight into these innovation efforts. Patents can be considered as an output measure for early-stage research [16], whereas clinical trials are a measure of output for later-stage research [17].

### 2.1. Data Collection and Curation

Patent data were gathered from the publicly accessible worldwide Espacenet database [18] on 27 January 2023. The demarcation of loop-mediated isothermal patents was achieved by searching for patents having assigned one of the following two cooperative patent classification (CPC) codes: C12Q1/68* or C12N9/1241*; and containing the phrases “*LAMP*” or “*loop-mediated isothermal amplification*” in the title, abstract, or claims. Per patent family number, the primary patent document was determined to be the document with the oldest application date. This resulted in an initial dataset of 3479 unique patents, based on the earliest filing data per patent family number.

This set included a disproportional amount of patents filed and published only in China, where patent filings are driven by motives other than innovation, such as government subsidies and tax benefits [19]. Moreover, China allows for patenting of traditional medicines, which are not patentable in other jurisdictions (Eland 2007). In line with other studies taking patent quality into account [20], patents filed by Chinese applicants that were only published in China were excluded from the dataset. This resulted in a set of 1134 unique patents.

Synonyms for patent applicants were subsequently harmonized to one name per applicant and recorded in a separate list. Applicants who were also mentioned as inventors (‘non-institutional applicants’) were removed as applicants because they are not considered market participants.

For clinical trials, a combined dataset was composed, consisting of data from the WHO ICTRP database as well as the U.S. National Library of Medicine (NLM) database on ClinicalTrials.gov [21,22]. These databases are regularly updated and contain records of clinical trials from 202 countries. The NLM database also includes completed clinical trials, thereby enabling us to provide a more reliable picture of trends over recent years. Searches were conducted on 30 May 2023 by searching for “LAMP” or “loop-mediated isothermal amplification”. Data were extracted to MS Excel and deduplicated. Clinical studies were only included when the following data were present: trial country, trial status, trial subject, sponsor, condition studies, trial phase, and registration date. The result was a list of 21 clinical trials.

### 2.2. Data Analysis

General lifecycle and geographic analyses were performed, followed by analyses of patent applicants, CPC codes, and patent claims. First, patent filing trends were examined. Annual accumulation of patent publications in a field of technology can indicate the general phase of development within that field on the technology saturation curve (S-curve), and this method is often used as a way of technological forecasting [23,24,25,26,27]. Second, patents were analyzed based on the jurisdictions for which patent protection was pursued, resulting in an overview of 5500 patent registrations. Many patents were published in several jurisdictions, and analysis thereof allowed for the depiction of a geographical heatmap of which markets are considered attractive by applicants. Third, patent applicants were analyzed according to applicant type (University/Research institution; Industry, SMEs/Large firms; Government/governmental organizations) and geographical location to get an understanding of where the most active stakeholders in the field are [20]. Fourth, a claim analysis was performed on the dataset, searching for specific pathogens in patent claims to classify patents per disease indication. Lastly, a CPC code analysis was performed as CPC codes are an excellent means for determining the state of the art in any given technology field. CPC codes were extracted, deduplicated, organized, and visualized into relevant groups/classification groups.

Data analysis was conducted on the clinical trial dataset. First, a general analysis was conducted to study the number of clinical trials per year. Second, the type of clinical trial sponsors and collaborators (research institute or industry) was defined. Third, clinical trial descriptions were read to extract the clinical conditions targeted in the trial so that they could be categorized into one of the following categories: viral, bacterial, parasitic, or fungal. Fourth, an overview in the form of a geographical heatmap was created revealing the locations of all included clinical trials. Lastly, the locations of the organizations acting as clinical trial sponsor or collaborator were analyzed.

## 3. Results

### 3.1. Life Cycle Phase

**Figure 1:**
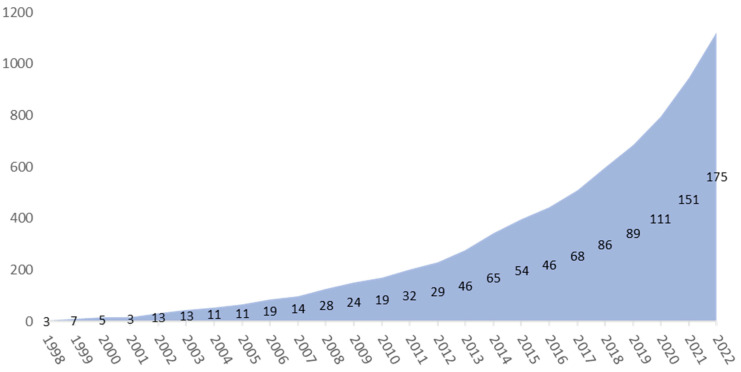
Cumulative patents of LAMP patents; in terms of innovation efforts (patents), LAMP technological development seems to be in the growth phase of the technology life cycle (S-curve) [23,27]).

With the first LAMP patent being published in 1998, the total number of patents grew to 1134 by the end of 2022. Figure 1 shows the cumulative number of LAMP patents over the years. The yearly increase in patents based on LAMP technology has steadily increased, with the last three years seeing more than 100 new LAMP-based patent applications. Considering the technology, S-curve LAMP technology developments seem to have entered a growth phase during the last decade.

With recent breakthroughs in CRISPR gene-editing technology, LAMP approaches may increasingly integrate it [28,29,30]. Since 2018, an increase in CRISPR-related LAMP patents has been noticeable, with the Broad Institute being the most active applicant. Successively, 8, 11, 19, and 15 CRISPR-related LAMP patents have been filed in 2019, 2020, 2021, and 2022, respectively (results not shown in the figure, at the time of the search, not all patents filed in 2022 had been published).

### 3.2. Patent Jurisdiction

**Figure 2:**
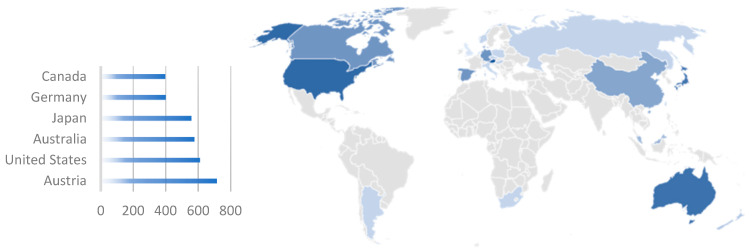
Jurisdiction of unique patent family filings. The intensity of the blue shading correlates with the number of patent filings, where darker shades represent a higher number of filings.

Figure 2 displays the jurisdiction where the unique patents in our dataset have been filed. Most patents are registered in several countries. In total, 5500 patent registrations have been filed in 25 different countries. Analysis of the jurisdictions revealed that most patents were filed in Austria, followed by the United States, Australia, and Japan. Further analysis revealed that patent protection was predominantly sought in high-income countries (based on the classification used by the World Bank): 20 high-income countries and 5 upper-middle-income countries.

### 3.3. Patent Applicants

**Figure 3:**
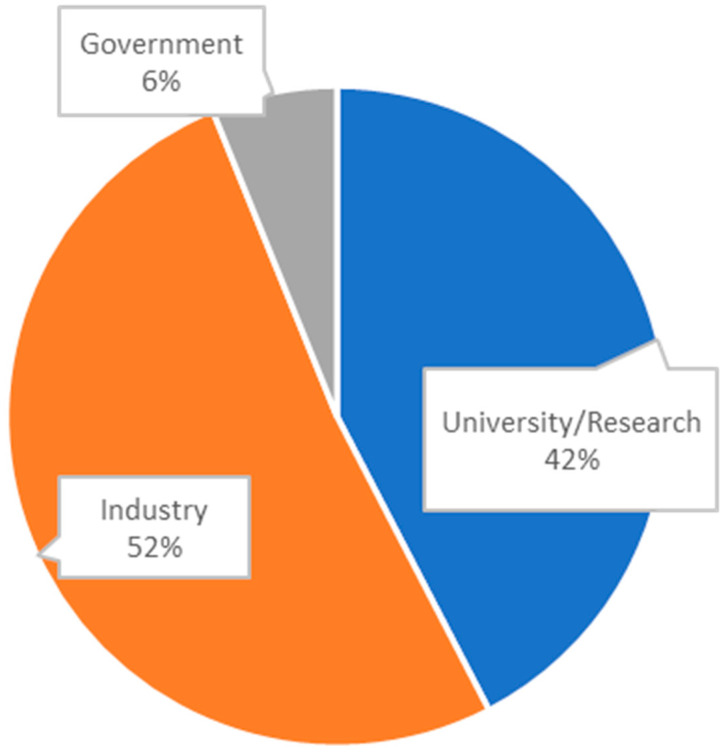
Overall distribution of applicant types; most applicants are either companies (industry) or universities/research institutions (including academic hospitals).

The applicants of patents are mostly industry participants (52%) and universities or research institutions, which includes academic hospitals (42%) (Figure 3).

The top 20 companies and universities or research institutions holding the most LAMP patents are listed in Table 1. Most of the top industry participants have been invested in this technology for at least a decade (e.g., EIKEN, TOSHIBA, Becton Dickinson, Qiagen, Sysmex), whereas others have built a strong IP position in more recent years (e.g., Alveo, Raytheon, Mammoth). Most of the research and technological development seems to be conducted by American universities or institutions during the past five to ten years. This indicates a strong academic interest in further development of the technology.

**Figure 4:**
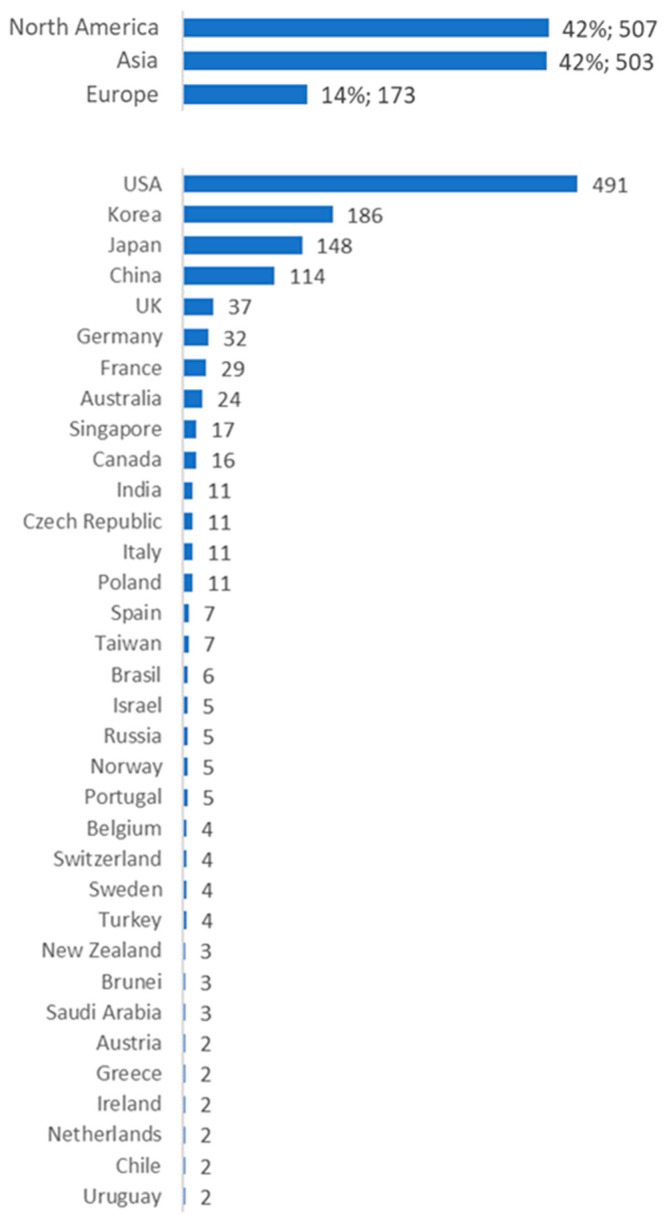
The number of patents by applicants per country; most technological development is conducted by American applicants (40%), followed by Asian applicants (36%) from South Korea, Japan, and China; and much less development seems to be taking place in Europe (14%).

US applicants are overrepresented, with 40% of patents having US applicants. A second group of overrepresented applicants originate from South Korea, Japan, and Asia, and they are responsible for another 36% of LAMP patents. A substantial portion of technological advancements in the field of LAMP techniques thus appears to originate from non-European countries. When combining the contributions of all European nations, the collective output places the European continent in third position in the patent filing ranking, constituting 14% of all patents related to LAMP techniques. This figure is considerably lower than that of the United States and even pales in comparison to South Korea as an individual nation. Figure 4 shows a breakdown of patents by continent and country of the applicant.

### 3.4. Clinical Trials

**Figure 5:**
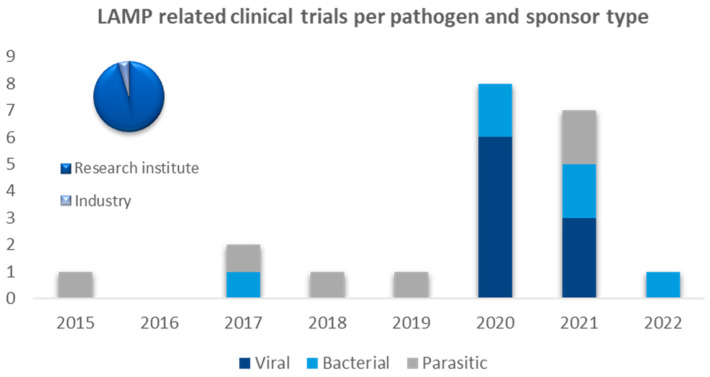
LAMP-related clinical trials performed by research institutes and industry, split per pathogen type.

Figure 5 shows the total number of LAMP-related clinical trials (21) performed by research institutes and industry, split per pathogen type (parasite, virus, bacteria). No LAMP-related clinical trials targeting fungal infections were identified. European institutions (35) were the most frequent sponsors and/or collaborators of clinical studies, followed by African (10), North American (6), South American (4), and Asian (1) institutions. Six clinical studies targeted parasitic diseases such as malaria (2x), leishmaniasis (2x), and trypanosomiasis (2x); nine clinical studies targeted viral infections (predominantly COVID-19), and six studies targeted bacterial infections including *Streptococcus* (1x), *Klebsiella pneumoniae* (1x), meningitis (1x), *Mycobacterium tuberculosis* (3x), and *Treponema Pallidum* (1x). Only one company appeared to be actively involved as a sponsor or collaborator in a clinical study. Furthermore, a substantial increase in LAMP-related clinical studies has occurred since the COVID-19 pandemic.

**Figure 6:**
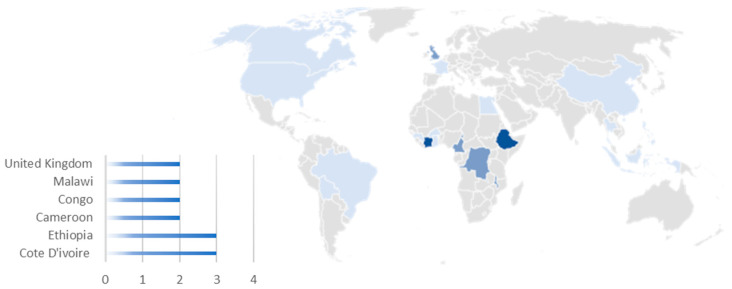
Geographical distribution of clinical trials. The intensity of the blue shading correlates with the number of clinical trials, where darker shades represent a higher number of clinical trials.

Figure 6 reveals the geographical distribution of the 21 clinical trials in 19 different countries. There appears to be a high concentration of clinical trials in resource-limited settings in the African continent (10 unique trials taking place in 16 African countries). These clinical studies seem to focus primarily on parasitic and bacterial diseases (five and four, respectively). In high-income regions, clinical trials predominantly targeted COVID-19 (eight trials on COVID-19 of which six were in high-income countries).

### 3.5. Patent Claims

Of the 278 unique patents targeting pathogens, 72% (200) patents targeted viral pathogens in their patent claims, 34% (95) targeted bacterial pathogens, 11% (31) parasitic pathogens, and 10% (29) targeted fungal infections.

**Figure 7:**
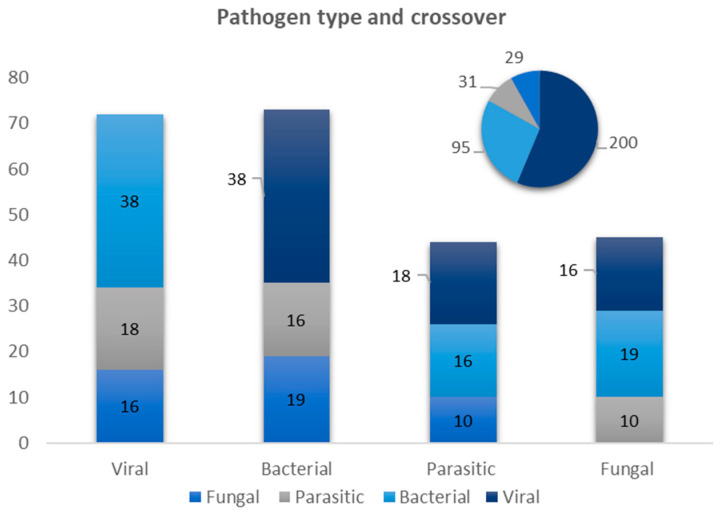
The number of patents per pathogen type frequency and how often pathogen crossover occurs within patents.

Figure 7 reveals a substantial crossover between pathogen types in patent claims. Patents describing viral pathogens in their claims (200) also appear to target bacterial pathogens in 19% of the cases and parasitic pathogens and fungal pathogens in 9% and 8% of the cases, respectively. Patents targeting bacterial pathogens (95) also appear to target viral pathogens quite frequently (40%) but less so for parasitic pathogens (17%) and fungal pathogens (20%). Patents targeting parasitic pathogens (31) seem to target both viral pathogens (58%) and bacterial pathogens (52%) quite often but fungal pathogens (32%) less frequently. Lastly, patents targeting fungal infections (29) very often seem to target bacterial pathogens (66%), followed by viral pathogens (55%) and parasitic pathogens (34%).

Ten unique patents seemed to include all four types of pathogens within their patent claims. Five patents included a combination of viral, bacterial, and parasitic pathogens, and another five patents included a combination of viral, bacterial, and fungal pathogens in their claims. No patents were identified focusing on a combination of fungal or parasitic pathogens in their claims without also including bacterial or viral pathogens.

**Figure 8:**
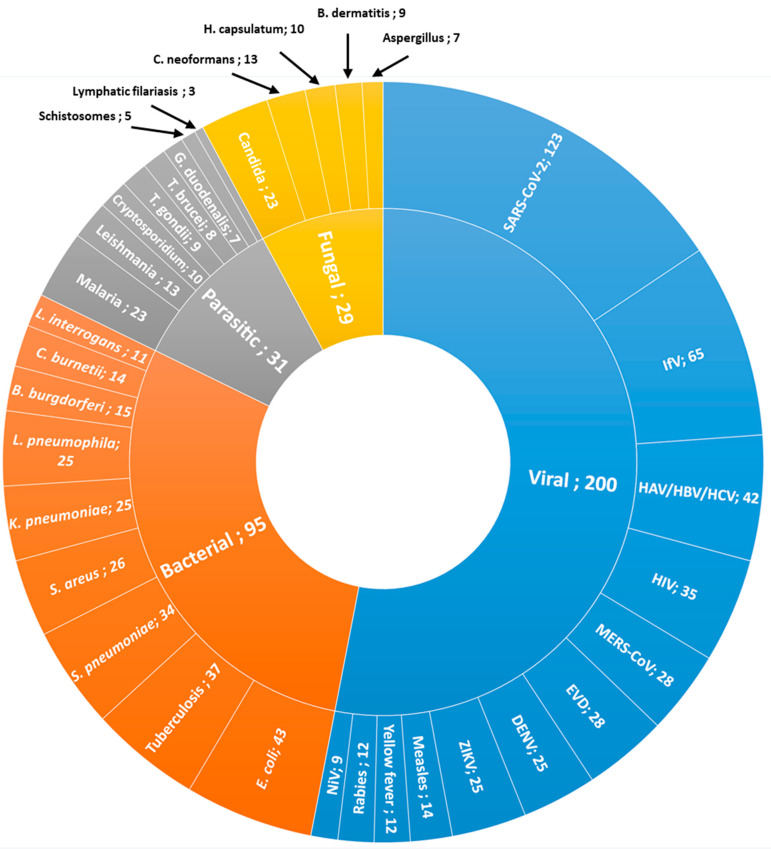
Frequency of pathogen types mentioned in the patent claims of unique patents.

Figure 8 further shows findings from the patent claim analysis and reveals the frequency of pathogen types mentioned in the patent claims of unique patents. Viral pathogens were by far the most frequently targeted in patent claims: 12 different viral pathogens were mentioned a total of 418 times in 200 unique patents. For bacterial pathogens, nine different species were mentioned a total of 231 times in 95 unique patents. Lastly, eight different parasitic pathogens were mentioned 78 times in 31 unique patents, and five fungal pathogens were mentioned 62 times in 16 unique patents.

COVID-19 was by far mentioned most often in patents specifically targeting pathogens. Of all unique patents specifically targeting pathogens, COVID-19 was mentioned in 45.7% of the cases, followed by the influenza virus at 24.2%, and hepatitis at 15.6%. For the “big three” of infectious diseases, tuberculosis was targeted in 13.8%, HIV in 13.0%, and malaria in 8.6% of “pathogen-targeting patents”. Relatively few patents seemed to target (neglected) parasitic or viral pathogens common in tropical regions, such as rabies, yellow fever, Leishmania, lymphatic filariasis, and schistosomes, for which there appear to be relatively few LAMP-related patents.

### 3.6. Cooperative Patent Classifications

**Figure 9:**
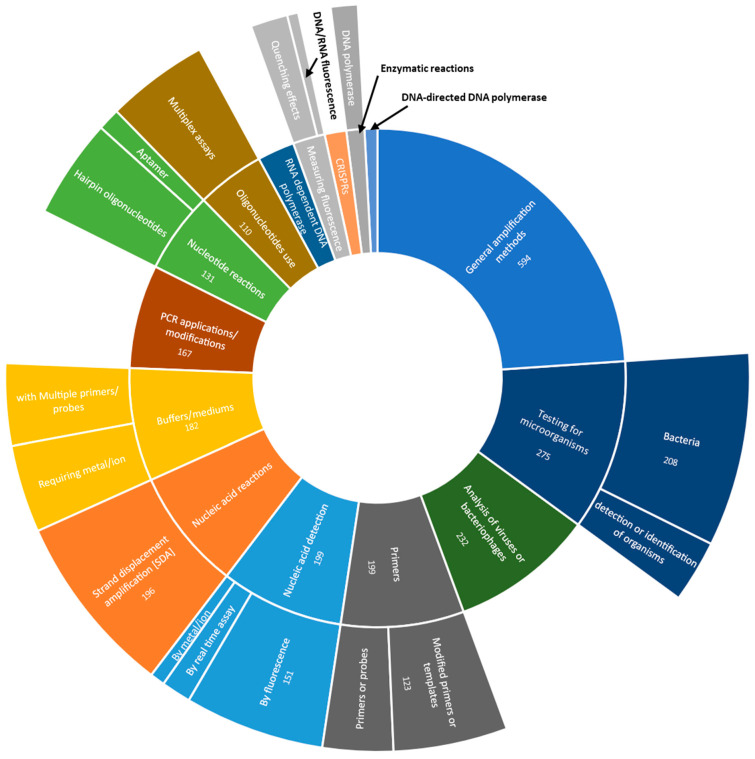
A display of the predominant CPC codes for LAMP diagnostic patents as an indication for the type of technology described within the patent, including the of patents in our dataset that were assigned these CPC codes.

Figure 9 shows the predominant CPC codes for LAMP diagnostic patents as an indication for the type of technology described within the patent. More than half ((52%, 594) Patents are generally assigned multiple CPC codes, which causes overlap of patents in this analysis) of patents in the dataset include the code describing general nucleic acid amplification techniques that do not fall under a specific group (type). In total, 17% (196) of patents include the code for reactions of nucleic acids characterized by strand displacement amplification (SDA); other types were much less prevalent or relevant. Another 24% (275) of patents were assigned codes describing measuring or testing processes for detection or identifications of organisms, in particular bacteria (18%, 208), whereas 20% (232) of patents included codes describing methods that are specifically designed for the analysis of viral nucleic acids or for the analysis of nucleic acids of bacteriophages. Classification codes describing specific types of nucleic acid detection were associated with 18% (199) of the patents, of which most (13%, 151) concerned detection characterized by fluorescence. Also, 18% (199) of the patents included codes related to primers, of which most (11%, 123) concerned methods using modified primers of templates. Finally, 15% (167) of patents were assigned the code describing applications dealing with modifications or improvements of PCR.

## 4. Discussion

Despite huge successes, the RT-PCR technique has several shortcomings in its application during sudden outbreaks and in low-resource settings, where shortages in specialized requirements can emerge with increased demand. This study shows that LAMP is an upcoming and promising alternative in the future of rapid testing of pathogens, especially in resource-limited settings. This study further exposes an interesting discrepancy between a focus of clinical research on unmet diagnostic needs for bacterial and parasitical tropical diseases (e.g., malaria, leishmaniasis, tuberculosis) in resource-limited settings, and a focus of basic research and commercial efforts (patents) on global viral indications such as SARS-CoV-2 and influenza with high earning potential. Finally, the study shows that European stakeholders are more concerned with the societal impact of technology, focusing on applications to meet unmet needs in resource-limited settings, rather than leading in innovation. On the other hand, American and Asian organizations seem to concentrate more on research, pioneering advancements, and commercial exploitation of the technology.

LAMP technology shows much promise as there seems to be increasing growth in patent activity in recent years, indicating a transition into a growth phase in the technology life cycle. As a means of technology forecasting [23,24,25,26], the life cycle analysis shows we are to expect a continuing growth phase in the coming years, followed by a maturity phase. Furthermore, the technology life cycle measured in cumulative patents usually precedes the life cycle of products based on the respective technology, which means a growth in clinical trials and subsequent product registrations is to be expected as well. This makes LAMP an important and promising alternative for rapid testing in the near future. Interestingly, the transition into the growth phase for patent activity is occurring twenty years after the development of the original LAMP method by Notomi et al. (2000) [5], patented by Eiken Chemical Co. Ltd., Tokyo, Japan, suggesting that the expiration of these patents may have relieved other developers from a license fee burden and potentially allowed for freedom to operate [16]. Coincidentally, the initial patents for Recombinase Polymerase Amplification (RPA) have recently expired [31].

In addition, this transition coincided with the recent pandemic and the high global need for rapid testing. Considering aforementioned limitations of the RT-PCR method, currently established as the “gold standard” technique for DNA amplification and rapid testing of COVID-19 [2,3,32,33], many researchers and innovators were highly motivated to work on alternative diagnostic methods. Our findings indeed indicate that SARS-CoV-2 was claimed in the patents most frequently, further indicating that much technological development was in part motivated by recent outbreaks.

LAMP has been discussed in existing literature as a promising alternative that is more economically viable and executable in low-resource laboratory settings [3,4,34,35,36]. The potential application of LAMP technology for Point of Care (PoC) diagnostics in more resource-limited settings and low-income countries is further exemplified by the substantial number of clinical studies targeting (neglected) tropical diseases, and most trials being carried out in low- and middle-income countries in Africa. However, it remains unclear why the adoption and expansion of this technology have not been more pronounced in these regions over the preceding two decades.

In literature, LAMP techniques have been proposed as a solution to address inadequate diagnostic capacities for high-burden diseases such as HIV, malaria, tuberculosis, and neglected parasitic diseases like leishmaniasis and trypanosomiasis [37]. Our results confirm a substantial focus on LAMP techniques for diagnosing (neglected) parasitic and bacterial infections. Interestingly, this focus is less evident in early-stage research and patenting efforts. Relatively few patents seem to target (neglected) viral or parasitic pathogens common in tropical regions, such as rabies, yellow fever, leishmaniasis, lymphatic filariasis, and schistosomiasis (only 31 out of 1134 unique patents were identified targeting parasitic infections). Conversely, early research and patenting efforts predominantly concentrate on global viral pathogens with higher profitability, commonly found in both high-income and low-income countries, such as COVID-19 and influenza. The reduced interest in early-stage research and patenting for parasitic infections may be attributed to low profitability and limited intellectual property protection in affected regions. Therefore, while there is a clear unmet need for diagnostics in neglected tropical diseases (NTDs), and the potential of LAMP techniques to address this need is high, it does not appear to have accelerated early-stage research and patenting activities targeting these kinds of pathogens. Exploring the development and implementation of favorable local business models, such as promoting regional manufacturing, might offer a way to enhance research efforts targeting neglected tropical diseases (NTDs).

Finally, it appears that European stakeholders prioritize societal impact and implementation of LAMP technology in resource-limited settings over commercial exploitation in high-resource settings. This is supported by our findings showing that European stakeholders are overrepresented in clinical trials that focus on tropical diseases in low- and middle-income regions. Conversely, Figure 4 shows that American and Asian applicants account for more than 75% of patent activity, leaving Europe lagging far behind when it comes to LAMP-related basic research, innovation, and technological development. As most of the patents are focused on diagnostics for SARS-CoV-2 and other globally prevalent pathogens, we can conclude that American and Asian stakeholders prioritize applications with higher future earning potential. It is difficult to come to a comprehensive explanation that could underpin this stark difference, as multiple elements may play a role. It could, for example, be generally associated with known commercialization gaps between Europe and North America [38], sometimes described as the ‘European Paradox’—EU countries playing a leading global role in terms of top-level scientific output but lagging behind in commercial exploitation thereof [39]. Europe traditionally concerns itself more with responsible and sustainable development of technology, considering factors such as social impact, ethical considerations, and environmental sustainability, exemplified by many of Europe’s policy frameworks and public funding programs [40,41,42]. In contrast, the US is often associated with prioritizing development and commercial exploitation of technology to drive economic growth and competitiveness. The findings mirror previous comprehensive analyses of Ebola patents, which showed that US and Asian applicants typically addressed self-centric unmet needs, whereas European applicants focused more on fulfilling altruistic needs [43]. Defourny and Nyssens [44] further discuss convergences and divergences regarding conceptions of social entrepreneurship between Europe and the United States. However, such general divergences insufficiently explain the difference between European and American applicants in this study, setting aside the notable activity of applicants from mainly Korea, Japan, and China. Perhaps further qualitative research could provide insights into the prioritization of societal impact by European stakeholders in this regard.

## 5. Implications and Further Research

This study implies a rather optimistic future for the application of LAMP technology in rapid diagnostic testing. It is expected that the future rapid diagnostic testing landscape will transition to faster and cheaper alternatives to be implemented closer to the patient or at point-of-care (POC), which are especially more relevant in resource-limited settings [45]. Rapid diagnostic testing has for a long time, and even during the recent COVID-19 pandemic, been dominated by a centralized lab-based testing infrastructure. Further development and innovation in this field may provide promising opportunities for the entrepreneurial community (researchers, founders, and investors) as well as policy advisors and decision-makers. Moreover, the adoption and implementation of these innovations will have implications for health policy, particularly relating to pandemic preparedness [46,47], in healthcare settings, and for global health in resource-limited settings [45,48].

As the field is transitioning towards the implementation of technologies enabling POC testing, it will be interesting to monitor innovations and technologies that will dominate the future of rapid diagnostic testing. In this context, further research may concern additional variables relating to funding and innovation efforts. This may include examining funding streams towards LAMP technology-related projects from national and international government bodies (public) and from foundations and NGOs (private). Furthermore, activity in the entrepreneurial community could be studied, which may concern activity regarding commercial (startup) companies focusing on applications of LAMP technology as well as venture capital investments in LAMP and other diagnostic technologies [26]. Another avenue for further research may focus on understanding barriers and facilitating factors in the adoption and implementation of LAMP technology in existing healthcare structures and health policy, as well as in the context of pandemic preparedness [46,47]. Such technology adoption research requires a more qualitative approach, especially relating to health policy and healthcare settings [49,50]. Lastly, the relevance of LAMP technology and similar rapid diagnostic testing technologies in resource-limited settings may also be further explored in the context of global health [45,48].

With ample avenues for further research, this study of the patent and clinical trial landscape of LAMP technology provides a first step in analyzing trends in rapid diagnostic testing. It shows that LAMP is an upcoming and promising alternative for rapid testing of infectious pathogens.

## Figures and Tables

**Table 1:** The top 20 companies and top 20 universities or research institutions as patent applicants.

Company (Industry)	Number of Patents	Year of First Patent	University/Research Institution	Number of Patents	Year of First Patent
Toshiba	24	2007	Broad Inst Inc	19	2015
Eiken Chemical	17	2006	Massachusetts Inst Technology	19	2015
New england Biolabs	13	2013	Harvard College	18	2014
Alveo Tech	11	2017	California Inst of Techn	16	2014
Becton Dickinson	10	2011	Univ California	15	2011
Raytheon Technologies	10	2022	Univ Sungkyunkwan Res & Bus	15	2013
Mammoth Biosciences	9	2020	Purdue Research Foundation	12	2022
Qiagen	9	2003	Univ Arizona State	9	2017
Sysmex	9	2004	Univ Texas	9	2003
Genomtec	7	2021	Korea Advanced Institute of Science and Tech	7	2018
Nippon	7	2011	Kyungpook Nat Univ Ind Acad	7	2014
Talis Biomedical	7	2018	Nat Inst Forest Science	7	2017
3M Innovative Properties	6	2016	Nat Univ Chungbuk Ind Acad Coop Found	7	2018
Capitalbio	6	2012	Univ Dankook Cheonan Campus Ind Academic Cooperation Foundation	7	2017
Mmonitor	6	2016	Univ Washington	7	2012
Speedx	6	2007	Univ Chung Ang Ind	6	2011
Dnaform	5	2006	Univ Hallym Iacf	6	2014
Moth Diagnostics	5	2021	Univ Pennsylvania	6	2012
Nanostring Technologies	5	2017	Univ Tsinghua	6	2012
Singlera Genomics	5	2018	Univ Korea Res & Bus Found	5	2020

## Data Availability

The raw data supporting the conclusions of this article will be made available by the authors on request.
